# Parental harsh parenting and non-suicidal self-injury among Chinese adolescents: the role of emotional uncontrollability, deviant peer affiliation, school disengagement, and self-control

**DOI:** 10.3389/fpsyt.2025.1732342

**Published:** 2026-01-20

**Authors:** Jingjing Li, Jiaqin Wang, Yue Tian, Danlin Cui, Xiaomin Xu, Qiongmei Zhang, Xingcan Ni, Chengfu Yu

**Affiliations:** 1 School of Health Management, Guangzhou Medical University, Guangzhou, China; 2 Department of Psychology/Research Center of Adolescent Psychology and Behavior, School of Education, Guangzhou University, Guangzhou, China

**Keywords:** adolescent, deviant peer affiliation, emotional uncontrollability, non-suicidal self-injury (NSSI), parental harsh parenting, school disengagement, self-control

## Abstract

**Background:**

Parental harsh parenting poses a significant risk for adolescent non−suicidal self-injury (NSSI). This longitudinal study investigated whether emotional uncontrollability, deviant peer affiliation, and school disengagement mediate the link between harsh parenting and adolescent NSSI, and whether these pathways are moderated by adolescents’ self-control.

**Method:**

A total of 513 Chinese adolescents (*Mage* = 10.32 years; *SD* = 0.96 years) were assessed at two time points six months apart.

**Results:**

T1 parental harsh parenting positively predicted T2 emotional uncontrollability, deviant peer affiliation, and school disengagement. T2 emotional uncontrollability and deviant peer affiliation, in turn, were associated with T2 NSSI. The mediating effect of T2 emotional uncontrollability on the relationship between T1 harsh parenting and T2 NSSI was significant. Moreover, this indirect link was significant for adolescents with low self-control, but not for those with high self-control.

**Conclusion:**

These findings highlighting the critical role of emotional uncontrollability as a mediator and, more importantly, self-control as a key moderator that buffers this risk pathway. These results suggest that interventions aimed at enhancing self-regulation and emotion-management skills may help mitigate the risk of NSSI among adolescents exposed to harsh parenting.

## 1 Introduction

Non-suicidal self-injury (NSSI) is defined as the behavior where individuals deliberately and repeatedly harm their own body tissues in ways not socially accepted, without having suicidal intent (1). NSSI is not only regarded as a strong predictor of suicidal behavior (2) but also has become a public health issue of global concern due to its continuously rising incidence rate (3). A recent systematic review on NSSI behaviors among the Chinese population found that the estimated lifetime prevalence of NSSI among adolescents is 24.7% (4). Given that NSSI may have long-term negative impacts on the psychological development of adolescent, in-depth exploration of the mechanism of adolescent NSSI urgently requires the attention of psychological workers and educators.

### 1.1 Parental harsh parenting and adolescent NSSI

The family level is the most significant ecological risk factor for adolescent NSSI (5). Parental acceptance-rejection theory (6) posits that compared to children raised with positive parenting styles, individuals who perceive themselves as rejected are more likely to experience mental health-related issues such as problem behaviors (7). Parental harsh parenting refers to harsh behaviors, attitudes, and emotions directed at children, with specific manifestations including physical aggression (e.g., slapping), verbal aggression (e.g., yelling), psychological aggression (e.g., neglect), and coercive/controlling behaviors (8). The hostility and neglect exhibited by parents during harsh parenting, combined with the influence of social learning, leave children unable to feel warmth and acceptance within the family, making them prone to a range of problem behaviors (9), including self-injury (10).

Compared to Western parents, Chinese parents tend to adopt an “authoritative parenting style” and express emotions more implicitly, which may constitute a unique family risk factor for NSSI among Chinese children (11). Liu et al. (12) conducted a two-year longitudinal survey of 373 junior high school students and found that harsh parental parenting styles were positively correlated with NSSI after 24 months. Results from a 15-year longitudinal survey of Canadian children and adolescents showed that experiencing parental harsh parenting in childhood leads to higher suicidal ideation during adolescence (13). More recently, longitudinal studies continue to underscore the long-term mental health risks associated with harsh parenting (10, 14).

According to the integrative model of self-injury, the family is a distal factor for NSSI. High-risk families (e.g., those involving traumatic experiences, childhood abuse) may cause individuals to develop internal or interpersonal vulnerabilities (such as poor emotion regulation), resulting in ineffective responses to life stressors and thus leading to NSSI tendencies or an increase in NSSI behaviors (15). This model has been widely recognized in self-injury-related research (16, 17). In summary, this study intends to explore the relationship between parental harsh parenting and NSSI, as well as the underlying mechanism.

### 1.2 The mediating role of emotional uncontrollability

Sense of uncontrollability is the individual’s negative subjective perception of their own control ability at the psychological level, including environmental uncontrollability, tool uncontrollability, and emotional uncontrollability (18). Emotional uncontrollability is a state in which an individual subjectively feels unable to moderate their emotional responses. This state may be caused by an overly intense emotional response, and it may further trigger a loss of control over behavior. According to Nock’s integrated theoretical model of NSSI (15, 19), harsh parenting (as a distal risk factor) is accompanied by the generation of negative emotions leading to emotional uncontrollability (proximal vulnerability) and individuals may regulate their emotional experiences through NSSI (functional behavior). Therefore, emotional uncontrollability may mediate the effect between harsh parenting and NSSI. Furthermore, according to emotional security theory (20), individuals assess their emotional security by perceiving parental interaction patterns and parenting behaviors within the family system. Under harsh parenting, individuals’ emotional stability and controllability are disrupted, leading to problem behaviors (21, 22). Harsh parenting affects emotional controllability both via the distal risk-proximal vulnerability pathway and by undermining family emotional security. And it exacerbates emotional dysregulation and ultimately increases the risk of NSSI. Parental conflict and harsh parenting often co-occur (23), and the research indicated that emotional uncontrollability mediates the relationship between parental conflict and NSSI (24). This study showed that exposure to parental conflict during childhood has a significant positive impact on NSSI in adolescent among some Chinese students. Existing literature has indirectly tested the mediating effect of emotional uncontrollability between harsh parenting and NSSI.

Authoritarian and harsh parenting styles are associated with adolescents’ emotional controllability (25). Because parents are the first teachers in the adolescent’ growth process. They influence children in all aspects of life, including values, emotions, and behaviors. To avoid punishment and under pressure, adolescents may develop emotional avoidance strategies such as “lying and evasion”, which may lead to emotional dysregulation. In contrast, children under gentle parenting tend to have better emotion regulation, and their physical and mental health as well as academic abilities develop more favorably. A study suggests that harsh parenting styles by fathers or mothers increase the likelihood of adolescents’ emotional uncontrollability (26).

Secondly, uncontrollable emotions may increase the risk of NSSI. Individuals with emotional dysregulation struggle to cope with negative emotions in a healthy way. They have low tolerance for negative emotions and are prone to immediate relief impulses due to emotional overload; they may use NSSI to achieve emotional relief and validation (27). Research shows that people with poor emotional controllability are more likely to engage in NSSI (28). Therefore, this study proposes Hypothesis 1: Emotional uncontrollability mediates the relationship between harsh parenting and NSSI.

### 1.3 The mediating role of deviant peer affiliation

During adolescence, peer relationships and social interactions play a pivotal role in development. Deviant peer affiliation refers to the extent to which individuals associate with peers who engage in maladaptive behaviors such as smoking, fighting, or alcohol use (29, 30). A substantial body of research has consistently demonstrated that deviant peer affiliation is significantly associated with a range of adverse outcomes in adolescence, including depression (31), Internet gaming disorder (32), and aggressive behavior (33). Collectively, these findings underscore the detrimental impact of deviant peer affiliations on adolescent psychosocial adjustment.

Within the family system, parenting styles are strongly associated with children’s psychological and physical development (34). Harsh parenting may deprive adolescents of adequate emotional support and behavioral reinforcement, fostering oppositional attitudes and significantly increasing their propensity to affiliate with deviant peers (35). From the perspective of social learning theory, when parents use harsh parenting, adolescents may internalize negative parent-adolescent interaction patterns and transfer these maladaptive behaviors to peer relationships (50). These dysfunctional interpersonal patterns can hinder the development of healthy peer connections, thereby heightening adolescents’ vulnerability to integration into deviant peer groups (36). Moreover, empirical research consistently demonstrates that adverse parent-adolescent interactions, such as parent-adolescent conflict, parental rejection, and childhood psychological maltreatment, are robustly linked to adolescents’ engagement in deviant peer affiliations (36–38).

According to deviance regulation theory, adolescents who affiliate with deviant peer groups are subject to group norms, which increases their tendency to conform their verbal and behavioral expressions to those of other group members (39). Moreover, self-injurious behaviors have been shown to exhibit peer contagion effects (40). Consequently, exposure to peers who engage in self-injury significantly elevates the risk that adolescents will engage in similar behaviors. On the other hand, deviant peer affiliation exposes adolescents to multiple stressors (41). When adolescents lack adequate psychological resources and effective coping strategies to regulate such stress, they may resort to maladaptive coping mechanisms, including NSSI (42). A growing body of empirical evidence supports this association, consistently identifying deviant peer affiliation as a significant risk factor for adolescent NSSI (43–45).

A growing body of research has examined NSSI through the integration of environmental and individual-level factors (46–48). However, less attention has been devoted to understanding NSSI within a multi-systemic ecological framework. The present study investigates adolescent NSSI by simultaneously considering family environmental influences and school-based peer dynamics, thereby contributing to a more comprehensive understanding of the developmental contexts underlying NSSI in adolescence. Based on the above theoretical and empirical foundations, we propose the following Hypothesis 2: Deviant peer affiliation mediates the association between harsh parenting and NSSI.

### 1.4 The mediating role of school disengagement

Schools represent a pivotal context in adolescent development and play a critical role in shaping youths’ psychological and behavioral adjustment (34). School disengagement refers to the erosion of an individual’s connection to the school system and encompasses both behavioral withdrawal (e.g., truancy, incomplete homework) and emotional detachment that arises from unmet needs within the school environment, such as inadequate academic support or conflictual teacher-student relationships (49). From the perspective of social learning theory, early parent-adolescent interaction patterns are internalized as internal working models (50), which subsequently influence behavioral and emotional regulation across multiple developmental contexts, including family and school settings. When adolescents are exposed to harsh parenting practices, they may internalize these maladaptive patterns and generalize them to peer interactions. Consequently, adolescents who display negative or aggressive behaviors toward peers are at heightened risk of peer rejection, which in turn may contribute to disengagement from school. Empirical research further demonstrates that distinct dimensions of parenting are differentially associated with adolescent school engagement (51). Notably, adverse parenting practices, such as insufficient parental monitoring and the use of corporal punishment, are consistently linked to lower levels of school involvement (52).

According to self-determination theory (53), external environmental factors that undermine the satisfaction of basic psychological needs within the self-system may lead individuals to experience persistent negative feedback, resulting in a deficit in adaptive coping strategies and increasing the propensity to engage in extreme regulatory behaviors. In the school context, adolescent disengagement is characterized by diminished feelings of belonging and a lack of perceived support, which may erode motivation to adhere to institutional expectations and heighten vulnerability to maladaptive behaviors, including aggression and rule violations (54). Consistent with this, empirical findings demonstrate that higher levels of school engagement are associated with reduced likelihood of aggressive conduct and problematic mobile phone use among adolescents (55, 56). Grounded in this theoretical and empirical framework, Hypothesis 3 is proposed: School disengagement mediates the association between harsh parenting and NSSI.

### 1.5 Self-control as a moderator

While the aforementioned multiple mediation model delineates potential pathways from harsh parenting to NSSI, individual differences in resilience are expected to significantly alter the strength of these associations. Among the most critical protective factors is self-control—conceptualized as an individual’s intrinsic ability to actively regulate behavioral responses, emotional states, and cognitive processes to achieve long-term goals (57). Contemporary extensions of the strength model of self-control (58) suggest that while harsh parenting depletes self-regulatory resources through chronic stress exposure, adolescents with high trait self-control demonstrate superior resource management and conservation strategies, thereby serving as a crucial buffer against familial adversity (14).

Empirical evidence increasingly supports self-control’s moderating function in developmental psychopathology. Self-control likely buffers the initial impact of harsh parenting on core mediators. Adolescents with heightened self-control possess enhanced capacity for cognitive reappraisal and impulse inhibition, preventing the escalation of transient distress into clinical-level emotion uncontrollability beliefs (59). Neurodevelopmental evidence suggests these adolescents exhibit strengthened prefrontal circuitry supporting emotion regulation (60), potentially weakening the harsh parenting-emotion uncontrollability beliefs link. Regarding deviant peer affiliation, adolescents with robust self-control demonstrate reduced impulsivity in social seeking behaviors, enabling more deliberate peer selection despite familial stress (61). Contemporary research indicates this protective mechanism remains significant even after accounting for peer network characteristics (62). Furthermore, self-control facilitates maintained school engagement amid adversity through sustained attention allocation and goal prioritization (63). A three-wave longitudinal study by Xiang et al. (64) specifically identified self-control as protecting academic engagement in Chinese adolescents experiencing family stress.

Self-control appears to buffer the progression from mediators to NSSI. Even when experiencing significant risk exposure, high self-control provides a crucial barrier against self-injurious acts through enhanced response inhibition and distress tolerance (65). Research examining the ideation-to-action framework for NSSI specifically identifies self-control deficits as critical in translating emotional pain into self-injurious behavior (66). In Chinese contexts, Jiang et al. (67) found self-control weakened the association between peer stress and NSSI frequency, highlighting its protective role at this advanced risk stage. Integrating the mediating and moderating mechanisms, the present study tests a comprehensive longitudinal moderated mediation model. We examine the mediating roles of emotional uncontrollability, deviant peer affiliation, and school disengagement, while positioning self-control as a critical moderator that is expected to buffer these indirect pathways. Accordingly, this study proposes the following hypotheses:

Hypothesis 4: Self-control moderates the indirect link between harsh parenting and NSSI through emotion uncontrollability beliefs.

Hypothesis 5: Self-control moderates the indirect link between harsh parenting and NSSI through deviant peer affiliation.

Hypothesis 6: Self-control moderates the indirect link between harsh parenting and NSSI through school disengagement.

## 2 Methods

### 2.1 Participants

In this study, a cluster random sampling method was used to select participants from four primary schools in Guangdong Province, all of whom were students from the fourth to sixth grades, and a two-wave longitudinal study was conducted. At Time 1 (T1), participants completed measurements including parental harsh parenting, NSSI, and demographic information (i.e., gender and age). At Time 2 (T2), participants completed measurements including NSSI, emotional dyscontrol, deviant peer affiliation, school engagement, self-control. The final dataset included 513 valid questionnaires, among which 275 were from males (53.6%), 238 from females (46.4%), with a mean age of 10.32 years (*SD* = 0.96 years; range from 9 to 13 years).

### 2.2 Measures

#### 2.2.1 Parental harsh parenting

The Harsh Parenting Questionnaire, compiled by Wang (8), was used to measure the harsh behaviors, emotions, and attitudes perceived by adolescents from their parents. This scale consists of 8 items (e.g., “When I do something wrong, my father loses his temper with me and even shouts at me”), with a 5-point scoring system where 1 represents “never” and 5 represents “always”. The higher the score, the more severe harsh parenting experienced by the adolescent. In this study, the Cronbach’s alpha for this questionnaire was 0.84.

#### 2.2.2 NSSI

This questionnaire was adapted by Yu et al. (68) based on the Deliberate Self-Harm Inventory compiled by Gratz (69), and 6 NSSI behaviors were selected for measurement. These six NSSI behaviors are the most common among Chinese adolescents, including cutting oneself, carving words or patterns on the skin with sharp objects until bleeding, severely scratching oneself until bleeding or scarring, pulling hair hard, biting oneself, and vigorously rubbing the skin until bleeding. Adolescents were asked to report the frequency of engaging in the above self-injury behaviors without suicidal intent in the past six months. The items used a 5-point rating scale, where 0 indicates “0 times” and 5 indicates “five times or more.” The higher the average score of the items, the more NSSI behaviors the individual had. NSSI was measured at both T1 and T2 in this study, with the Cronbach’s alpha for this questionnaire being 0.87 and 0.90, respectively.

#### 2.2.3 Emotional dyscontrol

The Beliefs About Emotions Questionnaire compiled by Manser et al. (70) was used to measure emotional dyscontrol. This scale consists of 9 items (e.g., “When I am upset, that feeling completely takes over my mind”), with a 5-point scoring system where 1 represents “strongly disagree” and 5 represents “strongly agree”. The higher the score, the higher the level of emotional dyscontrol of the adolescent. In this study, the Cronbach’s alpha of this scale was 0.92.

#### 2.2.4 Deviant Peer Affiliation

The Deviant Peer Affiliation Scale adapted by Yu et al. (71) was used to measure deviant peer affiliation. Participants were asked, “How many of your friends have engaged in deviant behaviors—such as fighting, smoking, and deliberately hurting themselves”. This scale consists of 8 items, with a 5-point scoring system ranging from 1 (*none*) to 5 (*six or more*). The higher the score, the more deviant peer affiliations the individual has. In this study, the Cronbach’s alpha of this scale was 0.69.

#### 2.2.5 School disengagement

The School Engagement Scale compiled by Wang et al. (72) was used to measure school disengagement. The scale includes three dimensions: cognitive, emotional, and behavioral, with a total of 23 items (e.g., “Daydreaming in class, not paying attention and listening carefully.”). The items used a 5-point scoring system where 1 represents “always” and 5 represents “never”. The higher the score, the higher the degree of school disengagement. In this study, and the Cronbach’s alpha of this scale was 0.90.

#### 2.2.6 Self-control

The Chinese version of the Self-Control Scale, compiled by Tangney et al. (73) and revised by Tan and Guo (74), was used to measure self-control. The scale includes 19 items (e.g., “I procrastinate things for so long that it affects my health or efficiency”). The items used a Likert 5-point rating scale, ranging from 1 (*strongly disagree*) to 5 (*strongly agree*). The higher the score, the higher the level of self-control. In this study, the Cronbach’s alpha of this scale was 0.73.

### 2.3 Procedure

This study has been approved by the Ethics Committee of Guangzhou University (GZHU202351), and informed consent has been obtained from the parents and students. Trained psychology research assistants assisted the participants in completing the self-report questionnaires, and the entire process lasted approximately 30 minutes. The anonymity of the participants was guaranteed, and they could withdraw from the study at any time without giving a reason. As a small token of gratitude, participants were given a commemorative pen after they finished filling out the questionnaires.

### 2.4 Statistical analysis

First, SPSS 27.0 was used for descriptive statistics and correlation analysis of the data. Second, Model 4 and Model 58 in the SPSS macro program PROCESS by Hayes (75) were used to test the mediating effect and moderating effect. The identified interaction effects were further examined using simple slope analysis. The significance of these effects was assessed via a bootstrapping procedure with bias correction, employing 5,000 bootstrap samples. A effect was considered significant if the 95% confidence interval (CI) excluded zero. Furthermore, the covariates of gender, age, and NSSI at T1 were included in all models.

## 3 Results

### 3.1 Descriptive analyses

Correlation coefficients and descriptive statistics of study variables are presented in **Table 1**. Specifically, T1 harsh parenting was positively associated with T2 emotion uncontrollability beliefs, T2 deviant peer affiliation, T2 school disengagement, and T2 NSSI. In addition, T2 NSSI was positively correlated with T2 emotion uncontrollability beliefs, T2 deviant peer affiliation, and T2 school disengagement. Finally, T2 self-control was negatively related to these five variables.

**Table 1:** Correlations and descriptive statistics of the variables.

Variable	1	2	3	4	5	6	7	8	9
1. Gender	1.00								
2. Age	0.04	1.00							
3. T1 HP	0.11*	−0.08	1.00						
4. T1 NSSI	0.02	−0.03	0.20***	1.00					
5. T2 NSSI	0.05	−0.02	0.16***	0.45***	1.00				
6. T2 EUB	−0.05	−0.01	0.20***	0.25***	0.38***	1.00			
7. T2 DPA	0.14**	0.08	0.25***	0.20***	0.26***	0.23***	1.00		
8. T2 SD	0.16***	−0.01	0.24***	0.18***	0.22***	0.34***	0.24***	1.00	
9. T2 SC	−0.10*	0.06	−0.23***	−0.17***	−0.23**	−0.41***	−0.15***	−0.52***	1.00
*M*	−	10.33	1.65	0.22	0.20	2.07	1.25	2.05	3.65
*SD*	−	0.96	0.66	0.61	0.64	0.97	0.37	0.60	0.77

HP, harsh parenting; NSSI, non-suicidal self-injury; EUB, emotion uncontrollability beliefs; DPA, deviant peer affiliation; SD, school disengagement; SC, self-control; T1, time 1; T2, time2. *p < 0.05, **p < 0.01, ***p < 0.001.

### 3.2 Testing for mediation effect

**Figure 1:**
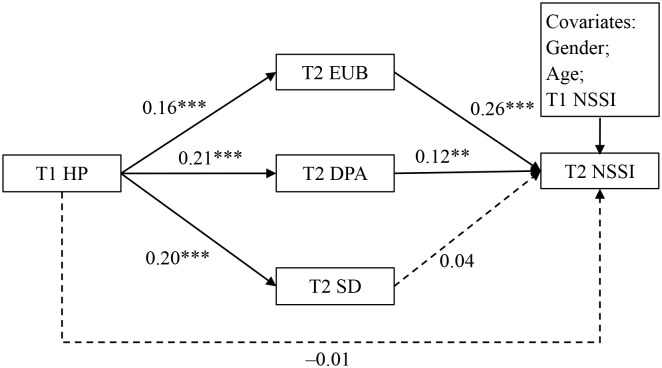
Testing the mediation effect of T1 harsh parenting on T2 NSSI. HP, harsh parenting; NSSI, non-suicidal self-injury; EUB, emotion uncontrollability beliefs; DPA, deviant peer affiliation; SD, school disengagement; T1, time 1; T2, time2. **p < 0.01, ***p < 0.001.

As **Figure 1** shows, after controlling for the covariates, T1 harsh parenting significantly and positively predicted T2 emotion uncontrollability beliefs, T2 deviant peer affiliation, and T2 school disengagement. Moreover, T2 emotion uncontrollability beliefs and T2 deviant peer affiliation were positively association with T2 NSSI. However, T1 harsh parenting and T2 school disengagement were not significantly associated with T2 NSSI. Further bootstrapping analyses showed that the mediating effect of T2 emotion uncontrollability beliefs (*Effect* = 0.04, 95% CI [0.01, 0.09]) on the relationship between T1 harsh parenting and T2 NSSI were significant; however, the mediating effect of T2 deviant peer affiliation (*Effect* = 0.02, 95% CI [–0.00, 0.06]) and T2 school disengagement (*Effect* = 0.01, 95% CI [–0.01, 0.03]) was not significant.

### 3.3 Testing for moderated mediation

**Figure 2:**
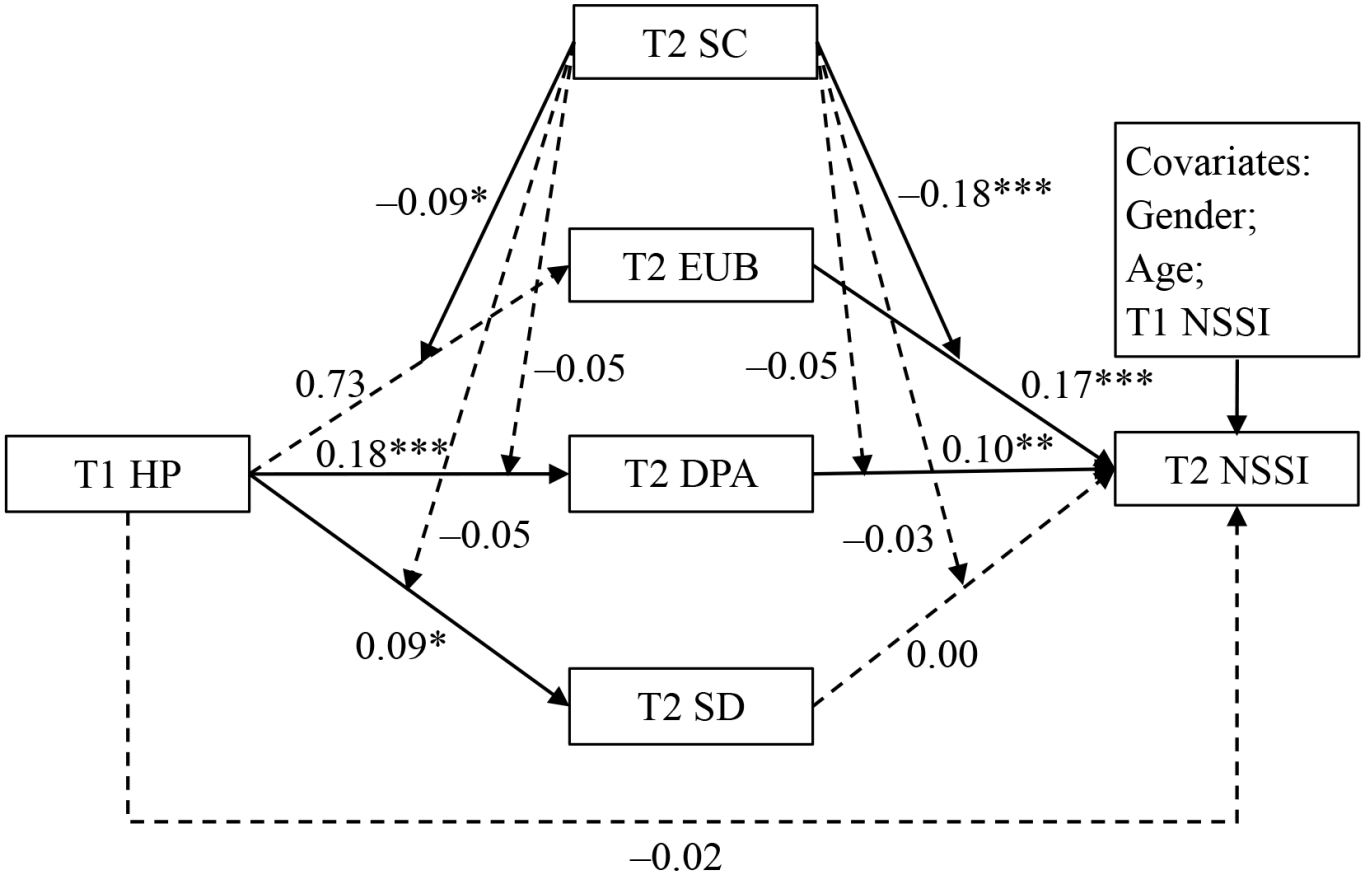
Testing the moderated mediation effect of T1 harsh parenting on T2 NSSI. HP, harsh parenting; NSSI, non-suicidal self-injury; EUB, emotion uncontrollability beliefs; DPA, deviant peer affiliation; SD, school disengagement; SC, self-control; T1, time 1; T2, time2. *p < 0.05, **p < 0.01, ***p < 0.001.

**Figure 3:**
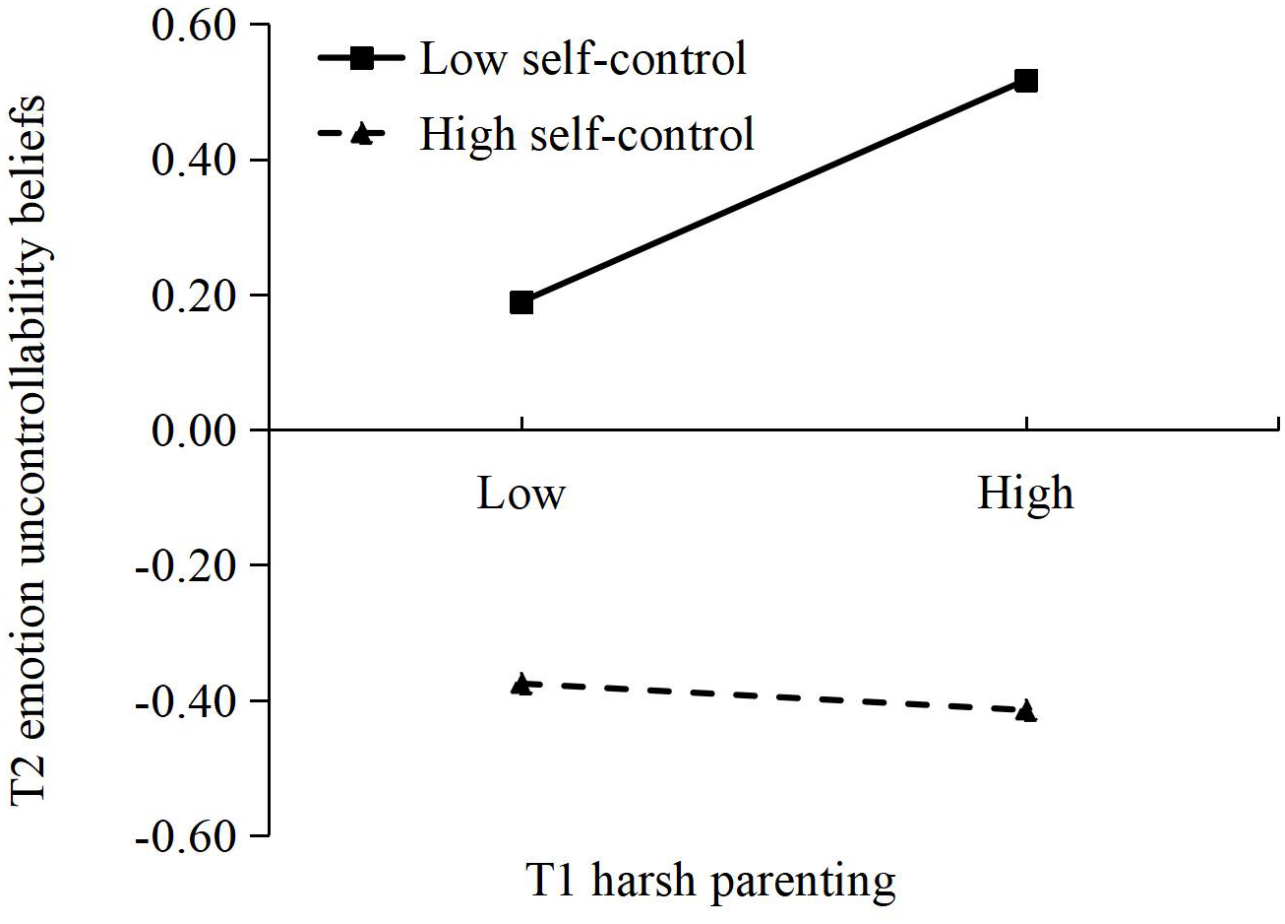
The moderating effect of T2 self-control on the relationship between T1 harsh parenting and T2 emotion uncontrollability beliefs.

**Figure 4:**
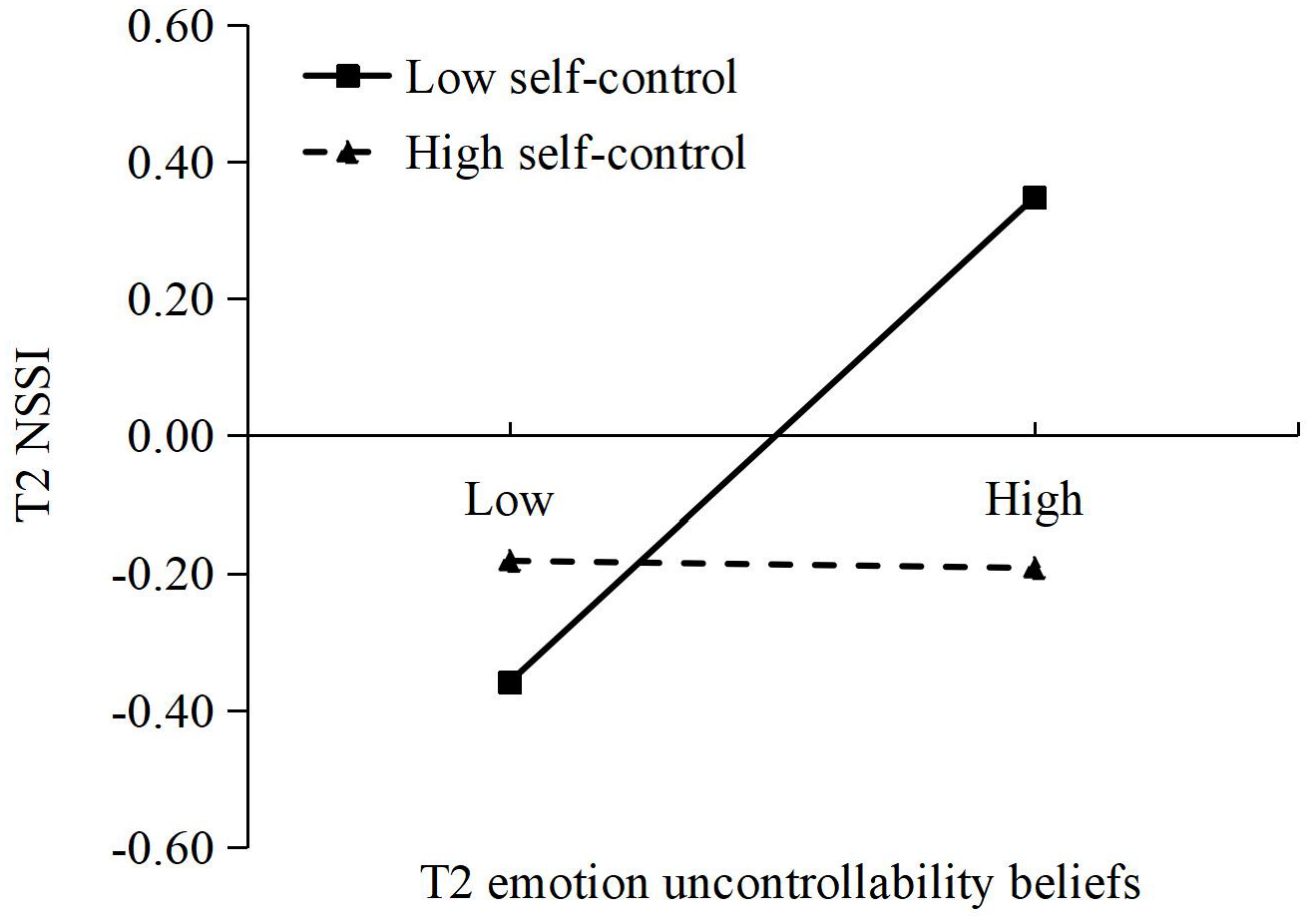
The moderating effect of T2 self-control on the relationship between T2 emotion uncontrollability beliefs and T2 NSSI.

As shown in **Figure 2**, T2 self-control moderated the link between T1 harsh parenting and T2 emotion uncontrollability beliefs, as well as the link between T2 emotion uncontrollability beliefs and T2 NSSI. Simple-effects analysis suggested that the relationship between T1 harsh parenting and T2 emotion uncontrollability beliefs was significant among adolescents with low self-control (*β* = 0.17, *p* < 0.01; see **Figure 3**), but not among adolescents with high self-control (*β* = –0.02, *p* > 0.05). Furthermore, the relationship between T2 emotion uncontrollability beliefs and T2 NSSI was also significant among adolescents with low self-control (*β* = 0.35, *p* < 0.001; see **Figure 4**), but not among adolescents with high self-control (*β* = –0.01, *p* > 0.05).

Overall, the indirect effect of T1 harsh parenting on T2 NSSI via T2 emotion uncontrollability beliefs was moderated by T2 self-control. For adolescents low in self-control, T1 harsh parenting had significant effect on T2 NSSI through T2 emotion uncontrollability beliefs (*Effect* = 0.06, 95% CI [0.01, 0.13]). In contrast, the indirect effect was not significant for adolescents high in self-control (*Effect* = 0.00, 95% CI [–0.01, 0.01]). Moderated mediation effects of T2 self-control were not found in the links between T1 harsh parenting and T2 NSSI through T2 deviant peer affiliation or T2 school disengagement.

## 4 Discussion

### 4.1 The mediating role of emotional uncontrollability

The finding showed that adolescents who experience T1 harsh parenting from their parents are positively correlated with T2 emotional uncontrollability, which may also increase the risk of transitioning to T2 NSSI. This finding supported that T2 emotional uncontrollability is a mediating mechanism between T1 harsh parenting and T2 NSSI.

According to Nock’s theory (15), emotional uncontrollability is an internal stressor that may lead to NSSI as a way to cope with the long-term effects of harsh parenting. Recent study found that harsh parenting-induced parent alienation and unhealthy family functioning are closely tied to a history of NSSI, with depressive symptoms and self-criticism amplifying this risk (76). Similarly, this result also aligns with emotional security theory (20), where adolescents with insufficient emotional security are unable to cope with negative emotional events and use NSSI to transfer pain, forming a vicious cycle.

Moreover, T2 emotional uncontrollability is positively correlated with NSSI. This finding is consistent with existing research (77, 78). Liu et al. (78) found that childhood maltreatment (such as emotional neglect or physical abuse) indirectly triggers NSSI through exacerbating emotional dysregulation--traumatic experiences disrupt the development of early emotional regulation, making individuals more prone to emotional uncontrollability → NSSI in adulthood.

### 4.2 The mediating role of deviant peer affiliation

The results revealed a positive association between T1 harsh parenting and T2 deviant peer affiliation, as well as between T2 deviant peer affiliation and T2 NSSI. However, T2 deviant peer affiliation did not emerge as a significant mediator in the relationship between T1 harsh parenting and T2 NSSI, providing no support for the hypothesis 2.

Specifically, the results reveal a positive association between T1 harsh parenting and T2 deviant peer affiliation, a finding consistent with prior empirical evidence (37, 38). As a maladaptive parenting style, harsh parenting not only contributes to emotional dysregulation and internalizing difficulties in children and adolescents (36), but also disrupts the development of social and interpersonal competence. Repeated exposure to negative parent-adolescent interactions may lead adolescents to internalize these dysfunctional relational patterns and generalize them to peer contexts (50). This process compromises their capacity to develop and sustain healthy peer relationships, thereby heightening their risk of affiliating with deviant peers (36).

Furthermore, the present findings reveal a significant positive association between T2 deviant peer affiliation and T2 NSSI. This observation is consistent with existing empirical evidence, which indicates that adolescents with higher levels of involvement in deviant peer networks are more likely to engage in NSSI (43, 45). Moreover, this association is theoretically supported by both deviance regulation theory and general strain theory, which posit that exposure to deviant peers and exposure to stressful life events may increase the likelihood of NSSI as a maladaptive coping mechanism in response to negative emotional states (39, 41).

However, T2 deviant peer affiliation did not significantly mediate the overall pathway between T1 harsh parenting and T2 NSSI. According to Nock’ s (15) integrative model, distal factors such as parental rearing practices and childhood trauma typically influence NSSI indirectly by shaping proximal factors, including emotional coping and stressful life events (48, 79). In the present study, both harsh parenting and deviant peer affiliation represent distal risk factors that operate at the same level of the individual’ s microsystem. Consequently, although both factors jointly contribute to adolescents’ risk for NSSI, harsh parenting does not exert its effect on NSSI through influencing adolescents’ engagement in deviant peer affiliation.

### 4.3 The mediating role of school disengagement

The results of the present study indicate that T1 harsh parenting significantly predicted T2 school disengagement. However, T2 school disengagement did not significantly predict T2 NSSI, nor did it significantly mediate the relationship between T1 harsh parenting and T2 NSSI. Therefore, Hypothesis 3 was not supported.

The finding that T1 harsh parenting predicts T2 school disengagement is consistent with prior empirical evidence linking negative parenting practices to increased risk of adolescent disengagement from school (52). This result aligns with social learning theory (50), which posits that adolescents internalize interpersonal interaction patterns experienced within parent-adolescent relationships and subsequently generalize these behavioral and emotional schemas to other social contexts. Notably, similar to how deviant peer affiliation often requires internal psychological factors (e.g., depression) to influence NSSI rather than directly mediating family-related pathways (45), school disengagement may also lack a direct mediating effect without the synergy of individual vulnerability factors. When these internalized patterns are characterized by hostility, control, or emotional unresponsiveness, they may impair adolescents ‘ peer relationships, increase susceptibility to peer rejection, and ultimately undermine engagement with school settings.

Furthermore, the present study revealed that T2 school disengagement did not significantly predict T2 NSSI, a finding that contrasts with prior empirical evidence. Notably, research among Chinese adolescents has shown that higher levels of school engagement are prospectively associated with lower rates of NSSI (80). As a central ecological context in adolescent development, the school environment serves as a critical source of emotional support and belongingness, both of which are fundamental to psychological well-being (53). When adolescents become disengaged from school and are consequently deprived of these essential psychosocial resources, they may be at increased risk for maladaptive coping responses, including self-injurious behaviors. The absence of a significant association in the current study suggests that the pathway from school disengagement to NSSI may be conditional, potentially moderated by unmeasured individual or contextual factors. Therefore, future research should aim to enhance sample diversity and integrate additional moderating or mediating mechanisms to better understand the complex interplay offactors underlying adolescent NSSI.

### 4.4 The moderating role of self-control

The findings of this study reveal a nuanced moderating role of self-control in the developmental pathway from harsh parenting to NSSI. Specifically, self-control significantly moderated both the initial link between T1 harsh parenting and T2 emotion uncontrollability beliefs, and the subsequent association between T2 emotion uncontrollability beliefs and T2 NSSI. These results partially support our hypotheses, highlighting self-control’s function as a critical buffer at distinct stages of the risk pathway.

The significant moderating effect observed in the relationship between harsh parenting and emotion uncontrollability beliefs provides compelling evidence for the protective role of self-control in adolescent development. This finding aligns robustly with the strength model of self-control (58), which conceptualizes self-regulation as a limited resource that can be depleted by chronic stressors like harsh parenting. Adolescents with higher levels of self-control demonstrate enhanced capacity for cognitive reappraisal and impulse inhibition when confronted with parental hostility, thereby attenuating the development of emotion uncontrollability beliefs (14). Furthermore, the identified buffering effect at the crucial stage between emotion uncontrollability beliefs and NSSI underscores self-control’s vital role in preventing the behavioral enactment of self-injury. Even when experiencing intense emotional turmoil, adolescents with robust self-control can better inhibit the impulsive urge to engage in NSSI as a maladaptive coping strategy (65). The dual protective function of self-control—both in mitigating the initial emotional impact of harsh parenting and in preventing the behavioral expression of distress—highlights its multifaceted importance in adolescent mental health. This pattern aligns with emerging evidence that self-control training interventions can effectively reduce NSSI frequency among at-risk adolescents (81). This pattern aligns with emerging evidence that interventions enhancing self-control can reduce risky behaviors in adolescents (65).

However, contrary to our hypotheses, self-control did not demonstrate significant moderating effects on the pathways involving deviant peer affiliation or school disengagement. This pattern of findings suggests that the protective influence of self-control may be particularly salient in managing the direct emotional consequences of harsh parenting and in preventing the translation of emotional distress into behavioral manifestations of NSSI (66). The non-significant moderating effects on peer- and school-related pathways indicate that these associations may be more robust and less influenced by individual differences in self-control, or that other contextual factors may play more prominent roles in these specific pathways (61). These findings have important implications for intervention strategies, suggesting that programs aimed at enhancing self-control may be particularly effective in helping adolescents manage emotional responses to harsh parenting and in preventing the transition from emotional distress to self-injurious behaviors.

### 4.5 Limitations and implications

This study provides empirical support for a longitudinal moderated mediation model linking parental harsh parenting to adolescent NSSI via emotional uncontrollability, with self-control acting as a critical buffer. Scientifically, these findings extend theoretical models by delineating a specific protective pathway through which self-control mitigates familial risk—not only by reducing emotional dysregulation but also by weakening the link between dysregulation and self-injury. Practically, the results highlight the need for multi-level interventions: parent-oriented programs should aim to reduce harsh disciplinary practices, while school- and community-based initiatives should integrate emotion-regulation training and targeted self-control enhancement for adolescents, especially those exposed to adverse family environments.

This study has some limitations. Firstly, all variables relied on adolescents’ self-reported data, which may be subject to subjective bias. It is difficult to objectively capture the actual parenting behaviors through self-reporting, which may affect the accuracy of the measurements. Second, the research sample come from Chinese adolescents, so caution should be exercised when generalizing the results to samples from other cultures. For example, Chinese culture emphasizes the unique parenting concept of strict discipline, which fundamentally differed from the Western parenting norm of opposing corporal punishment. Third, this study used two waves of longitudinal data with a 6-month interval to examine the association mechanisms between variables. It cannot completely rule out short-term interference from third variables, such as sudden emotional uncontrollability or deviant peer affiliation. Future research can use multi-wave longitudinal tracking designs to enhance the stability of research findings.

## 5 Conclusions

This study examined the longitudinal association between parental harsh parenting and adolescent NSSI, with a focus on the mediating roles of emotional uncontrollability, deviant peer affiliation, and school disengagement, as well as the moderating role of self-control. Results indicated that T2 emotional uncontrollability significantly moderated the relationship between T1 parental harsh parenting and T2 NSSI. Furthermore, the indirect effect of T1 parental harsh parenting on T2 NSSI through T2 emotional uncontrollability was moderated by T2 self-control. These findings underscore the adverse impacts of parental harsh parenting and emotional uncontrollability on adolescent NSSI, as well as the protective role of self-control. To prevent adolescent NSSI, parents and school-based practitioners should attend to the roles of parenting practices, emotion regulation capacities, and self-control skills. Interventions ought to prioritize enhancing adolescents’ emotion regulation, and self-control to promote their overall psychological and physical well-being. It should be noted that the reliance on self-reported data may introduce common method bias. Future research would benefit from multi-wave longitudinal designs incorporating multiple informants to better capture dynamic processes and strengthen causal inference.

## Data Availability

The raw data supporting the conclusions of this article will be made available by the authors, without undue reservation.
